# Live Attenuated Reassortant Vaccines Based on A/Leningrad/134/17/57 Master Donor Virus Against H5 Avian Influenza

**DOI:** 10.2174/1874285801711010316

**Published:** 2017-11-30

**Authors:** Irina Kiseleva, Natalie Larionova, Larisa Rudenko

**Affiliations:** 1 Institute of Experimental Medicine; St Petersburg, Russia; 2 Saint Petersburg State University, St Petersburg, Russia

**Keywords:** Highly pathogenic H5N1 avian influenza, Future pandemic, Live attenuated pandemic influenza vaccine, LAIV, H2N2

## Abstract

**Background:**

The H5N1 avian influenza was first recognized in humans in Hong Kong 20 years ago. Current enzootic spread of highly pathogenic H5N1 virus among wild and domestic poultry and a number of severe human respiratory diseases caused by this pathogen have stimulated necessity of development of potentially pandemic influenza vaccines.

**Discussion:**

In the past few years, significant research was conducted on how to prevent H5N1 influenza. Live, attenuated cold–adapted reassortant influenza vaccine (LAIV) is considered as one of the most promising candidates for pandemic and prepandemic vaccines. LAIV has proven to be safe and efficacious; pandemic LAIV might be more effective than inactivated vaccine in providing broader immune response.

**Conclusion:**

This review covers development of LAIVs against potential avian “pandemic” H5N1 subtype based on cold–adapted A/Leningrad/134/17/57 (H2N2) master donor virus backbone, and their preclinical and clinical studies.

## 1 INTRODUCTION

In recent times, the focus has mainly been placed on potential pandemic avian influenza viruses, which may acquire mutations facilitating their transmission to humans and subsequent human–to–human spread. One of the most potential pandemic pathogens is avian influenza virus subtype H5N1.

H5N1 viruses have circulated enzootically in wild birds and domestic poultry for years, persisting in Middle East Asia, Africa, and Europe. In 1997, highly pathogenic avian influenza A viruses (HPAIV) of the H5N1 subtype escaped the wild avian gene pool; the first laboratory–confirmed case of human infection with influenza H5N1 occurred in Hong Kong in a 3 year old boy who died from acute respiratory distress secondary to viral pneumonia [1].

**Fig. (1):**
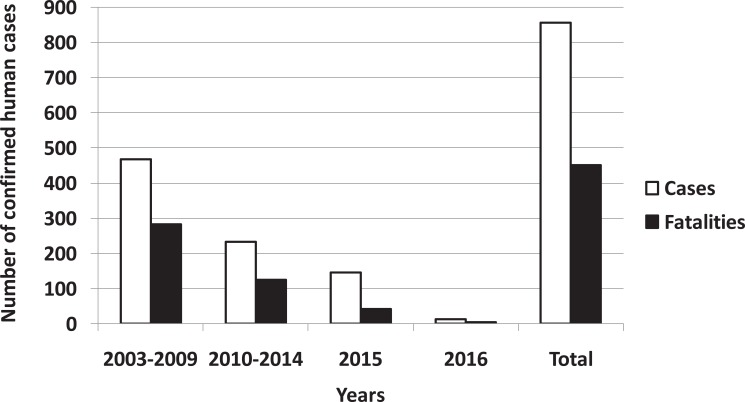
Cumulative number of confirmed human cases for avian influenza A(H5N1) reported to WHO, 2003–2016 (adapted from [2]).

A link between viral circulation in poultry and occurrence of human cases of H5N1 infection was confirmed. Since 2003, spillover from poultry to humans has caused a total of 856 documented human infections with avian influenza A(H5N1) virus, found in 16 countries with 452 fatalities to date (Fig. **1**) without adaptation to or enhanced transmissibility between humans.

Due to the high lethality and virulence, the highly pathogenic avian influenza virus (HPAIV) of subtype H5N1 is the world's largest pandemic threat. Continued transmission of avian influenza viruses from birds to humans presents an ongoing threat and underscores the urgent need for efficacious, cross–protective vaccines to prevent their spread among humans.

Current recommendation of WHO is that evolution of H5N1 avian influenza virus does not increase risk to public health [3], avian influenza H5N1 human cases remain rare and sporadic events; a number of confirmed cases is decreasing from year to year as it can been seen from Fig. (**1**). However, this virus still can serve as a progenitor to future pandemic virus. New subclades of H5N1 avian influenza viruses have been disseminated widely across the world. The probability of outbreaks of highly pathogenic avian influenza A virus of subtype H5N1 in human population is still high. It was conclusively proven in ferret model that avian H5N1 influenza viruses can acquire the capacity for airborne transmission between mammals and therefore constitute a risk for a new influenza pandemic [4-6]. Unprecedented spread between birds and humans of HPAIV might result in hundreds of human infections with fatal cases. This has highlighted the urgent need for the development of potential pandemic vaccines against avian influenza viruses.

In recent years, interest in live, cold–adapted, reassortant influenza vaccine (LAIV) has grown considerably, following the WHO’s recognition of the advantages of LAIV over inactivated vaccine (IIV) in the event of a pandemic [7, 8]. LAIV is administered by nasal spray. Intranasal administration does not require trained personnel; this makes it easier to use. Another major advantage of the LAIV vaccine over the standard shot with IIV is that it produces immunity similar to natural infection thus creating an early, long–term and broad immune response involving mucosal, humoral and cellular immunity without causing the disease. According to the WHO, there is evidence that in pandemic situation LAIV might be more effective than IIV [9]. Live, attenuated influenza vaccines may possibly induce a broader and more long–lasting protection than inactivated vaccines [10].

Russian live attenuated (*att*) influenza vaccine (LAIV) technology was developed at the Institute of Experimental Medicine (IEM), St Petersburg, Russia [11]. This vaccine has been approved and successfully used for over fifty years in millions of Russians – children, adults and the elderly [12-15]. The Russian LAIV is based on cold–adapted A/Leningrad/134/17/57 (H2N2) (Len–MDV) and B/USSR/60/69 Master Donor Viruses (MDVs), which are reassorted with circulating epidemic strains recommended by World Health Organization (WHO) for use as vaccine candidates in current influenza season.

As part of the WHO global action plan, IEM is generating a collection of LAIV strains against a number of potential pandemic viruses, including H5N1 subtype (Table **1**). In the current review development, preclinical and clinical studies of prepandemic LAIVs against H5N1 avian influenza viruses (H5–LAIVs) based on A/Leningrad/134/17/57 (H2N2) MDV are discussed.

## 2 BACKGROUND

### 2.1 Development of H5–LAIV Candidates on A/Leningrad/134/17/57 (H2N2) MDV Backbone

#### 2.1.1 Classical Genetic Reassortment Procedure

In 2007, the first Russian prepandemic LAIV candidate against H5 avian influenza, A/17/duck/Potsdam/86/92 (H5N2) (Len–dP), was developed on the base of nonpathogenic A/duck/Potsdam/1402–6/86(H5N2) strain which circulated in nature over 30 years ago [16]. Then, another prepandemic LAIV candidate, A/17/Vietnam/04/65107 (H5N2) (Len–VN), based on H5N1 virus of clade 1, A/Vietnam/1203/2004 was developed [17]. To date, these strains are immunogenically obsolete. For this reason, a potentially pandemic A/17/turkey/Turkey/05/133 (H5N2) (Len–tT) vaccine candidate related to more modern antigenic clade 2.2 was generated [17].

In brief, 6:2 reassortants between Len–MDV and wild–type virus are produced in embryonated chicken eggs following a number of rounds of selective propagation. The production and selection for reassortants is undertaken in the presence of anti–Len–MDV serum. Low temperature propagation (26 degrees C) is also used as selective factor, except for the initial cross (1:1 co–infection of Len–MDV and wild–type virus) and the last cloning by end–point dilution which are both carried out at 32 degrees C. This method of classical reassortment was successfully used for decades to develop seasonal 6:2 LAIV candidates [18, 19].

However, classical reassortment of the Len–MDV with A/duck/Potsdam/1402–6/86 (H5N2) resulted in the temperature sensitive/cold–adapted (*ts/ca*) H5N2 LAIV candidate, that contained the only HA gene segment from wild–type virus six (reassortant genotype 7:1).

**Fig. (2):**
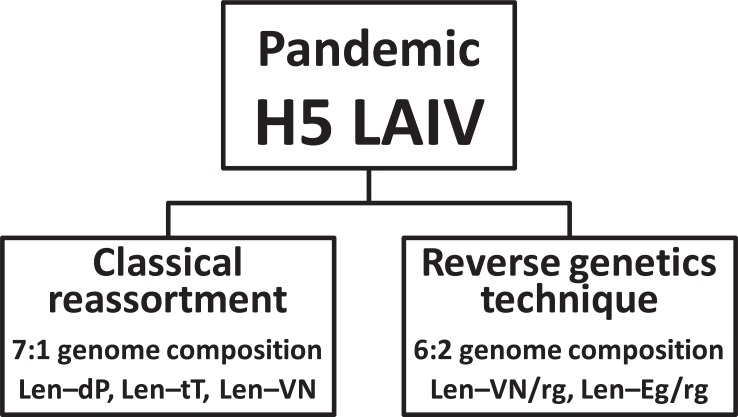
Two ways to develop live attenuated H5 pandemic influenza reassortant vaccine.

In the next experiments, to reduce a risk of manipulations with highly pathogenic for humans avian influenza viruses, PR8–based reassortants for parenterally administered, IIV of subtype H5N1, VN/PR/CDC–RG and NIBRG–23 were used as a source of external glycoproteins [17]. The HA of these H5N1/PR8 viruses is engineered by Reverse Genetics (RG) to remove four basic amino acid codons from the cleavage site of HA as described in [20], resulting in a virus that is considered to be attenuated for natural hosts and safe for humans. However, classical reassortment of the Len–MDV with H5N1/PR8 viruses is also resulted in 7:1 H5N2 viruses that contained six internal genes and NA gene of MDV (Fig. **2**).

Full–genome sequencing of 7:1 vaccine reassortants did not reveal significant changes, which can alter anticipated virus biological properties.

Interestingly, another group of authors faced a similar problem when trying to create 6:2 reassortants based on H5N1 viruses [21]. The results of their attempts were also reassortants with 7:1 gene segment ratio (genome composition). One of the possible reasons for the complexity of inheritance of avian virus origin NA in avian and human reassortant viruses may be associated with a higher body temperature of birds (40 – 42 degrees °С) and correspondingly higher temperature optimum for avian influenza virus polymerases. The problem in obtaining the desired 6:2 gene configuration may be also a result of co–infection of avian and human viruses. The functional incompatibilities between the viral proteins or RNA segments of two differing strains may lead to the segment mismatch that limits the reassortment efficiency [22-24].

Functional balance between HA and NA which is an important condition of influenza virus efficient replication, as well as a role of HA and NA specificities at oligosaccharide level in maintaining such balance, remains poorly studied. HA of avian origin binds exclusively and NA digests efficiently α2–3–sialylated carbohydrate chains, while HA of human origin interacts with α2–6 chains and low–active NA cleaves both α2–3– and α2–6–sialosides. Shtyrya *et al* [25] suggested that combination of avian HA and human NA may result in decreasing the replicative potential of reassortant virus because of disturbance of a functional balance between “alien” HA and NA. However, Larionova *et al* [17] demonstrated that combination of avian H5HA and human N2NA resulted in significant increasing the infectivity of 7:1 reassortants in compare with H5N1 parental viruses which contained both HA and NA of avian origin. In addition, phenotypical pattern of H5N2 LAIV candidates did not differ from such of the Len–MDV – transferring seven genes from Len–MDV into the H5N1/PR8 genome led to a dramatic decrease in infectivity of resulting H5N2 reassortants at the temperature of 38–39 degrees C (*ts* phenotype) and significantly increased their ability to grow at the temperature of 26^o^C (*ca* phenotype).

The authors suggested that in the case of generating a LAIV vaccine candidate for protecting humans against HPAIV, reassortants carrying the HA gene of pathogenic H5 virus and other genes from an attenuated MDV can be a good thing as they could provide extra level of safety for the LAIV candidate. The HA of H5N2 LAIV reassortants is modified for reduced virulence, but it has been observed that avian influenza NA may also be involved in the manifestation of the pathogenic properties of the virus [26]. The presence of NA of cold–adapted MDV, together with genetically modified H5–HA may provide an extra layer of safety for attenuation of H5N2 LAIV candidate against highly pathogenic avian influenza virus of H5N1subtype. As for the immune response, antibodies to the influenza virus HA are known to be the main component of the protection against human [27] and avian influenza viruses [28]. Thus, the evaluation of the individual contributions of each of the surface proteins to the induction of HPAIV–neutralizing serum antibodies and protective immunity showed that immunization of chickens with Newcastle disease virus expressing H5 hemagglutinin of avian influenza virus single or in combination with avian N1 neuraminidase caused in both cases 100% protection from challenge infection with H5N1 HPAIVs. The avian NA in the vaccine preparation did not improve protection generated by antibodies to H5 HA. Immunity to NA extended survival but did not prevent death from HPAIV challenge [29].

#### 2.1.2 Reverse Genetics Technique

Influenza virus reassortants can be also generated by plasmid–based reverse genetics from segments of DNA (Fig. **2**). Because avian–human H5N1 6:2 vaccine reassortants were not possible to obtain despite repeated attempts, alternative reverse genetics approach has been used. Reverse genetics is a promising technology, especially for development and construction of prepandemic influenza vaccines. MedImmune, a biotechnology company (USA), uses reverse genetics technology for development vaccine strains of seasonal, prepandemic and pandemic influenza vaccines as part of the current egg produced LAIV product.

Two H5N1 *ca* reassortant vaccine candidates, caVN1203–Len17rg (H5N1) (Len–VN/rg) [30, 31] and caEG321–Len17rg (H5N1) (Len–VN/rg) [31], were generated by plasmid–based reverse genetics, with six internal genes from Len–MDV and polybasic–cleavage site–deleted H5HA and intact N1NA genes from A/Vietnam/1203/2004 (H5N1) clade 1 or from A/Egypt/321/2007 (H5N1) clade 2.2 viruses, respectively. 6:2 H5N1–RG LAIV vaccine candidates were rescued by co–transfecting plasmids encoding HA and NA genes of H5N1 viruses with plasmids encoding six internal genes of Len–MDV, as described [30].

The 6:2 reassortant H5N1–RG LAIV viruses were fully sequenced and the absence of any unwanted spontaneous mutations and quasispecies was confirmed. Phenotypic analysis of 6:2 H5N1–RG LAIV reassortants indicated that the *ts/ca* phenotypes of the H5N1 reassortant viruses were consistent with *ts/ca* phenotype of Len–MDV.

### 2.2 Preclinical Testing of H5 LAIV Candidates on A/Leningrad/134/17/57 (H2N2) MDV Backbone

Studies on the attenuation, safety, immunogenicity, protective and cross–protective efficacy of H5 prepandemic LAIV candidates were conducted in different animal models (Table **2**).

#### 2.2.1 Mice and Guinea Pigs

A number of sensitive and convenient models are used to test for acute and sub–acute toxicity of drugs, vaccines and other immunobiological preparations. Guinea pigs and mice are the most commonly used species. These two species were used for determination of acute and sub–acute toxicity of Len–tT LAIV candidate [17]. Administration of Len–tT LAIV did not cause death, did not change the external appearance and behavior of animals, did not affect their consumption of food or water, and had no significant effect on body weight.

The results of histopathology study reveal that inoculation of Len–tT LAIV candidate did not cause in mice any inflammation, destructive or dystrophic changes in systemic organs and no dystrophic changes of neurons were observed in brain. The histopathology picture of samples of organs in vaccine groups was similar to that obtained from placebo group. Administration of H5 LAIV vaccine candidates did not cause gross morphological changes, suggesting positive safety profile of H5N2 LAIV [17].

Mice are the most convenient and widely used animal model for influenza vaccine research in terms of size, cost, husbandry requirements etc. This model was used for protective and cross–protective efficacy of Len–dP (H5N2), caEG321–Len17rg (H5N1) and caVN1203–Len17rg (H5N1) LAIV candidates. Immunization of mice with two doses of H5–LAIV candidates induced a strong immune response, and animals were protected against not only homologous but also heterologous highly pathogenic wild–type virus challenge [33]. Replication of challenge viruses in the upper and lower respiratory tracts of immunized animals was significantly reduced compared to the controls, and no signs of disease were observed in any of the vaccinated animals [30, 31, 33].

In summary, the vaccination of mice with Len–dP, caEG321–Len17rg (H5N1) or caVN1203–Len17rg (H5N1) LAIV candidates provided substantial cross–protection from challenge with H5N1 HPAIV.

#### 2.2.2 Monkeys

Monkeys are thought to more closely reflect the human response to influenza than more distantly related mammalian species (mice, ferrets, hamsters, guinea pigs etc). Thus, monkeys have been used to study HPAIV infection caused by avian H5N1 and virus [34]. However, their high cost, complex husbandry requirements, relatively low availability, and ethical issues make nonhuman primates less accessible for routine studies of influenza than another animal models. Despite these difficulties, it was important to characterize the first Russian prepandemic LAIV against H5 HPAIV in full. Thus, safety and cross–protection efficacy of Len–dP LAIV against a modern HPAIV were studied in Java macaques [4]. Monkeys were vaccinated with two doses of Len–dP LAIV; they were monitored for a week after each vaccination by examining body temperatures, behavior and weight loss. Len–dP LAIV was safe and areactogenic for monkeys. No fever or other side effects were observed following vaccination. After challenge with a modern heterologous HPAIV A/chicken/Kurgan/02/05 (H5N1) clade 2.2 half of vaccinated monkeys were shown to be fully protected. Duration and severity of fever reactions, the absence of viremia and virus replication in the upper respiratory tract were significantly reduced [5].

#### 2.2.3 Ferrets

For pandemic/prepandemic candidate influenza vaccine viruses against HPAIVs preclinical testing in ferrets and chicken should be performed [35]. The WHO strategy to demonstrate the absence of highly pathogenic characteristics includes, in particular, assessment of attenuation of the H5 LAIV candidates in ferrets and their non–pathogenicity in chickens.

Retention of key attenuating mutations is one of the very important characteristics of reassortant LAIV [36, 37]. Genetic stability of Len–dP LAIV after replication in upper respiratory tract of ferrets was confirmed. Vaccine viruses isolated from the immunized ferrets were shown to preserve all attenuating mutations described for the Len–MDV [16]. Ferret challenge study demonstrated reasonable protection of ferrets vaccinated with Len–dP LAIV from severe respiratory damage and death after challenge with heterologous HPAIV A/turkey/Turkey/1/05 (H5N1) clade 2.2 [38].

Three other intranasal LAIVs against pandemic influenza H5 variants, Len–tT (H5N2), Len–VN (H5N2) and Len–VN/rg (H5N1) were evaluated for attenuation, immunogenicity and efficacy in ferret challenge study [17, 30]. Ferrets were intranasally inoculated with H5 LAIVs to confirm their safety and attenuation properties. No H5 LAIV viral antigen expression was seen in any of the lung and nasal turbinate samples tested, while H5N1 HPAIV was detected in tested samples on day 3 and 5 post–inoculation. No clinical signs of illness including weight loss, dehydration, diarrhea, or dyspnea were seen in vaccinated animals. No histological changes typical for influenza virus infection were seen in the lungs of animals vaccinated with H5N2 LAIV candidates. Vaccinated ferrets did not demonstrate any reduction in body weight compared to placebo group.

H5 LAIVs protected ferrets from challenge with homologous HPAIV, while placebo–vaccinated animals demonstrated clear signs of disease and succumbed to infection. Infection with wild–type H5N1 influenza of which H5 was homologous to the vaccine induced severe disease in ferrets that were vaccinated with the placebo. The animals suffered from a strong reduced activity and heavy breathing, severe weight loss and severe fever and one animal died from the infection. In these animals, virus replication was detected at high titers in the throat, lung and trachea 5 days after infection. Upon post mortem examination, the lungs appeared swollen and showed multifocal consolidation. On the macroscopic level, approximately 70% of the tissue was affected. Microscopically, approximately 60% of the lung parenchyma were affected. Vaccination with H5–LAIV significantly reduced replication of challenge HPAIV in nasal turbinates and completely prevented its replication in lung tissue, while in placebo group wild–type H5N1 virus was detected in the nasal turbinates and lungs on day 3 and 5 post–inoculation. Vaccination significantly reduced all the infection related clinical signs and virus replication in the respiratory tract. Vaccinated ferrets showed minimal reduced activity, minimal weight loss and minimal fever. Moreover, none of the ferrets died from the infection. Virus replication was reduced to just above detection level in the throat and was below detection level in the trachea and lung. On the macroscopic level, no or minimal abnormalities were observed in the lungs, edema was not present. Microscopically, the infection damage was restricted to a minimal to light inflammation.

Two doses of caVN1203–Len17rg (H5N1) clade 1 LAIV elicited strong cross–reactive immune response; animals were protected from heterologous challenge with clade 2.2 HPAI viruses; a superior cross–protection of LAIV over IIV was demonstrated.

The H5–LAIVs raised specific antibodies against H5 influenza virus in significantly high titer, especially after a second dose. In addition, immunological cross–reactivity of H5–LAIV candidates was demonstrated. The results indicated that H5–LAIVs elicited a cross–reactive antibody response to the H5N1 viruses of clades 1 and 2.2. In contrast, no cross–reactive antibody response to clade 2.3 virus was observed.

#### 2.2.4 Chickens

Infection of poultry with HPAIVs can cause severe disease with high mortality. Genes of reassortant H5–LAIV candidates coding for glycosylated surface proteins are inherited from avian influenza parental viruses – HA of 7:1 vaccine candidates or HA+NA of 6:2 vaccine candidates, respectively. Thus, evaluation of safety and attenuation of H5–LAIV candidates in chicken model is important to answer a question – do live attenuated vaccines based on HPAIV pose threat for the poultry industry?

Evaluation of safety and attenuation of H5–LAIV candidates was performed in White leghorns. For the determination of pathogenicity of H5–LAIV candidates, chickens were inoculated intravenously and observed daily for 14 days for clinical signs and death. To determine infectivity, chickens were inoculated intranasally. On day 3 post–infection oropharyngeal and cloacal swabs were collected from chickens and virus replication was assessed in embryonated chicken eggs. The chickens were observed for clinical signs of disease and death for 21 days, at which time serum samples were harvested and tested for presence of antibodies.

The intravenous pathogenicity index of tested viruses Len–dP [33], Len–VN, Len–tT and Len–VN/rg [17, 38] in chicken was 0. It means that no birds showed any clinical signs or died during the 10–day observation period after intravenous vaccine injection. On day 3 after intranasal administration H5–LAIV, vaccine viruses were not detected in swabs of the upper respiratory tract and of the cloaca, lungs, kidneys, heart and brain tissues. H5–LAIV candidates were unable to replicate productively in birds and released to the environment, being completely attenuated. None of birds presented antibodies detectable after intranasal administration.

These data suggest that H5–LAIV candidates can be used for manufacturing human prepandemic influenza vaccines against highly pathogenic avian influenza viruses with minimum threat posed to the poultry industry.

In conclusion, regardless of the gene ratio in vaccine candidate (7:1 or 6:2) LAIVs against pandemic H5 were attenuated, immunogenic and effective in protecting animals of different species from severe disease, mortality and pathology and almost completely reduced virus replication.

### 2.3 Phase I–II Clinical Trials of H5 LAIV Candidates on A/Leningrad/134/17/57 (H2N2) MDV Backbone

Two 7:1 prepandemic H5N2 LAIV candidates, Len–dP and Len–tT, have been chosen to be included in the Phase I–II clinical trials in healthy adult volunteers (Table **3**) [39, 40]. On the analogy with seasonal vaccines, prepandemic LAIV must be proven safe and protective. WHO has developed recommendations to assure the quality, safety, and efficacy of the vaccine for pandemic situations [36]. According to these recommendations, clinical trials of H5–LAIV candidates contained safety studies and assessment of immunogenicity. Safety studies included determination of adverse reactions, replication, shedding, transmission potential and genetic stability of the vaccine virus. For evaluation of immunogenicity antibody response, cell mediated response and cross–reactivity were determined. Volunteers in each group were given two doses of vaccine 21 days apart or two doses of placebo.

#### 2.3.1 Clinical Observations (Adverse Reactions)

Both vaccines were well tolerated and no clinically significant adverse events were observed. Any safety concerns were not indicated (Table **3**). Most local and general adverse events were temporary, mild to moderate in severity and self–limiting. Among vaccinees with Len–dP LAIV, the most common safety complaints were catarrhal symptoms (40%) [39, 41]. The most common adverse events reported following receipt of Len–tT LAIV included nasal congestion, sneezing, catarrhal nasopharynx, sore throat, fever, and chills and occurred at similar frequencies in vaccine and placebo recipients [40]. Importantly, that a few reactions reported in Len–dP trial were of short duration with no consequences; the number of solicited adverse events reported in volunteers vaccinated Len–tT LAIV were not statistically different from that of the corresponding placebo groups.

#### 2.3.2 Shedding

In total, Len–dP LAIV virus was recovered by culturing nasal swabs in eggs from 11/20 (55%) and 14/20 (70%) vaccinees after dose 1 and 2, respectively [39]. Detection of virus shedding by PCR was not performed. Len–tT LAIV virus was detected by PCR in nasal swabs in 28/30 vaccinees (93%) after the first vaccination and in 21/29 (72%) after the second vaccination. Virus from nasal swabs was recovered in embryonated eggs in 10/30 vaccine recipients (33%) after the first and 6 of 29 (21%) after the second dose.

The duration of shedding of Len–tT LAIV virus in adult volunteers was very restricted and limited with only one day. In contrast, the duration of shedding of Len–dP LAIV was substantially higher and continued for up to 6.6 and 6.3 days after dose 1 and 2, respectively.

#### 2.3.3 Genetic Stability

The possibility of acquisition of additional mutations in LAIV virus genome during its replication in embryonated chicken eggs or humans is not surprising. Theoretically, each passage of an influenza virus may introduce new mutations or revert attenuated virus to virulent form. From this point of view, evaluation of the possibility of reversion to partial or full virulence post vaccine administration to people and genetic stability of LAIV candidate are extremely important quality characteristics. From safety issues first of all retention of attenuating mutations in internal genes known for MDV must be confirmed. Secondly, evaluation of stability of gene coding for HA is also very important, especially for prepandemic LAIV despite a fact that the development of prepandemic LAIV candidate involves modifications in the HA cleavage site [35].

According to WHO position on assurance of safety of LAIV, the absence of reversion to virulence (genetic stability) should be determined in preclinical and clinical studies [36]. Clinical trials in 20 adult volunteers confirmed genetic stability of the Len–dP LAIV [15]. Genetic and phenotypic stability of another H5–LAIV candidate, Len–tT after replication in humans was also demonstrated [42, 43]. A number of coding mutations in internal genes responsible for attenuation of Len–17 MDV and Len–17 MDV–based reassortant LAIVs is well known [44-46]. All these *att* mutations known for Len–17 MDV, were preserved in the genome of 16 Len–tT (H5N2) vaccine isolates recovered from nasal swabs of vaccinees. No mutations in HA which may lead to reversion to wild type virus HA were detected.

#### 2.3.4 Transmission

Since reassortant LAIV can replicate in upper respiratory tract of vaccinated individuals, there is a concern about a risk of unpredictable reassortment in nature between cold–adapted and wild–type viruses as a result of simultaneous infection of human host with vaccine virus and circulating wild–type influenza strain which might produce a progeny that contain novel, more virulent genotypes. However, no evidences of prepandemic Len–MDV–based H5 vaccine virus transmission from vaccinated individuals to their unvaccinated contacts have been reported. Of note, vaccine virus was not detected in placebo groups indicating the lack of person–to–person transmission.

#### 2.3.5 Immunogenicity

Clinical testing of prepandemic H5–LAIV candidates in healthy adult volunteers revealed comparable levels of their immunogenicity. After two doses of Len–dP LAIV about half of vaccinees demonstrated seroconversions in HAI and microneutralization tests [15]. After the second vaccination with Len–tT LAIV, 38% of the vaccine recipients presented a ≥4–fold increase in HAI test and 48% – in microneutralization assay, respectively. To further characterization of antibody response to Len–tT vaccine, several additional tests, including detection of serum IgG and IgA antibodies as well as mucosal IgA antibody, by ELISA were performed. Cumulative data on antibody immune responses showed that 79% of vaccinated subjects had antibodies after dose 1 and/or 2.

Serum immune responses are recognized as a good correlate of vaccine protection for inactivated influenza vaccine. In contrast, HAI titers do not appear to correlate with protection against influenza by LAIV [47]. WHO considers important to assess the potential efficacy of LAIV by measuring not only humoral response but also innate, mucosal and cellular immune responses [36].

Len–dP LAIV was able to induce reliable increases in T–cell levels. Two doses of Len–dP LAIV promoted CD4^+^ and CD8^+^ memory T–cell responses in peripheral blood of healthy volunteers [48, 49]. Similar results were obtained for Len–tT vaccine. Overall, 69% subjects vaccinated with Len–tT LAIV had ≥4–fold increases in CD4^+^ or CD8^+^ responses [40]. In total, percentage of all positive immunological reactions after dose 1 and/or 2 reached 97% for Len–tT LAIV. Interestingly, the low duration of Len–tT vaccine virus shedding did not influenced dramatically on its immunogenicity.

#### 2.3.6 Cross–Reactivity and Prime–Boost Strategy

 Influenza A (H5N1) viruses continue to undergo antigenic drift. During last decades 10 clades (0 – 9) and numerous subclades have been identified [50]. Information regarding the ability of vaccines from one clade of virus to stimulate antibody against other clades of virus is of critical importance. There are reasons to believe that H5–LAIV may elicit production of broadly reactive antibodies, which could neutralize the newly emerged avian influenza viruses. Rudenko et al [39] demonstrated that humoral immune responses to the Len–dP LAIV were cross–reactive, and near 30% of serum samples reacted with antigenically divergent virus A/Indonesia/05/2005 (H5N1).

A number of publications illustrated the possibility to enhance the immune response to inactivated and live influenza vaccines. These studies suggested that administration of a combination of IIV and LAIV may provide a strategy for improved influenza vaccination, in particularly – in elderly [51, 52]. The idea to administer of a combination of two different types of influenza vaccines led to the development of novel vaccination approach – so called prime–boost strategy, which could result in induction of significant immune response to a booster IIV following priming with homologous or heterologous LAIV [53-55]. Similar to these prime–boost studies, assessment of immune responses to tT (H5N1) inactivated influenza vaccine among individuals previously primed with Len–tT (H5N2) LAIV was performed [56]. Data revealed that priming with Len–tT LAIV induced a long–lasting B–cell immunological memory in subjects against antigenically related influenza virus, which was characterized by more prompt and vigorous antibody production to a single dose of H5–IIV given 18 months later.

Thus, prepandemic vaccines of 7:1 gene configuration demonstrated a good safety profile and were well tolerated. The two–dose immunization schedule resulted in measurable serum and local antibody production, and generation of CD4^+^ and CD8^+^ memory T cells. Dose–related increases in immune responses were demonstrated. Importantly, the H5–LAIV–induced antibodies were cross–reactive and were able to neutralize antigenically divergent viruses.

## 3 DISCUSSION

Preventive vaccination remains one of the principal weapons against most infectious diseases, including influenza. WHO experts recognize advantages of live, attenuated influenza vaccines over inactivated influenza vaccines especially in the pandemic situation; LAIV was included into WHO Global influenza pandemic action plan and WHO global influenza preparedness plan [8, 9].

As of today, the focus has mainly been placed on potential pandemic avian influenza viruses, which may acquire mutations facilitating their transmission to humans and subsequent human–to–human spread. One of most potential pandemic pathogens is avian influenza virus subtype H5N1. To this end preparing a National collection of vaccine strains against potentially pandemic influenza viruses, which may cause serious and fatal disease, and constructing the appropriate LAIVs is of strategic importance.

Much progress has been made since 1997 when the first laboratory–confirmed case of human infection with H5N1 HPAIV was confirmed. A number of candidates for live, attenuated influenza vaccine against H5 avian influenza viruses was generated on Len–MDV backbone by either method of classical reassortment or by plasmid–based reverse genetics; genome composition of resulting reassortants was 7:1 and 6:2, correspondingly.

Attenuated phenotype of H5 LAIV candidates was confirmed by molecular genetics and virological methods. All H5 reassortants retained temperature–sensitive, cold–adapted properties of Len–MDV, regardless of the gene segment ratio, 7:1 or 6:2.

Preclinical data strongly support the safety, immunogenicity and protective efficacy of prepandemic H5 LAIV reassortants. LAIVs against pandemic H5 HPAIV were immunogenic and effective in protecting from severe disease, mortality and pathology and almost completely reduced virus replication in different animal models. No significant difference in attenuation, safety, immunogenicity, protection and cross–protection efficacy of 6:2 or 7:1 vaccine candidates was found.

Clinical trials of two 7:1 prepandemic vaccines against H5N1 potentially pandemic viruses, А/17/turkey/Turkey/05/133 (H5N2) and A/17/duck/Potsdam/86/92 (H5N2) revealed their high safety and immunogenicity. In addition, antibodies induced by H5–LAIVs were cross–reactive and were able to neutralize antigenically divergent viruses. Moreover, administration of Len–tT LAIV primes the immune system and leads to a pronounced immune memory response after H5N1 IIV boost given 1.5 years later.

## CONCLUSION

As of today, intensive studies on improving traditional 6:2 LAIV by including additional NP or M1 proteins from wild–type virus rather than MDV are being conducted [57-59]. Despite this fact, 7:1 vaccine–appropriate reassortants might also be good candidates for LAIV in line with LAIV candidates with 6:2 or 5:3 genome composition.

## Figures and Tables

**Table 1:** List of prepandemic H5 LAIVs prepared on A/Leningrad/134/17/57 (H2N2) MDV backbone.

LAIV Candidate, Genome Composition	Wild–type Parental Virus	Generated by	Stage Study	Refs.
Len–dP (H5N2), 7:1^1^	A/duck/Potsdam/1402–6/86 (H5N2)	Classical reassortment	Phase I–II clinical trials completed. The vaccine is registered in Russia	[32, 33, 39]
Len–tT^2^ (H5N2), 7:1^1^	A/turkey/Turkey/1/2005 (H5N1), clade 2.2	Classical reassortment	Phase I clinical trial completed	[17]
Len–Vn^2^ (H5N2), 7:1^1^	A/Vietnam/1203/2004 (H5N1), clade 1	Classical reassortment	Preclinical studies completed	[17]
Len–Vn/rg^2^ (H5N1), 6:2^3^	A/Vietnam/1203/2004 (H5N1), clade 1	Reverse genetics	Preclinical studies completed	[30, 32]
Len–Eg/rg^2^ (H5N1), 6:2^3^	A/Egypt/321/2007 (H5N1), clade 2.2	Reverse genetics	Studies in mice	[31]

^1^Vaccine strain inherited only HA gene from wild–type parental virus and remaining 7 genes – from Len–MDV (7:1 genome composition). ^2^ΔHA, polybasic cleavage site deleted. ^3^Vaccine strain inherited HA and NA genes from wild–type parental virus and remaining 6 genes – from Len–MDV (6:2 genome composition).

**Table 2:** Preclinical studies of Russian prepandemic H5 LAIV candidates in animal models.

H5 LAIV Candidate	Animal Model	Study Subject	Refs.
Len–dP (H5N2)	Mice	Attenuation, immunogenicity, protective and cross–protective efficacy	[16, 32, 33, 37]
	Ferrets	Attenuation, genetic stability, cross–protective efficacy	[16, 37]
	Chicken	Attenuation	[33]
	Macaques	Safety, cross–protective efficacy	[15]
Len–tT (H5N2)	Mice	Acute and sub–acute toxicity tests	[17, 38]
	Guinea pigs	Acute and sub–acute toxicity tests	[17, 38]
	Ferrets	Safety, attenuation, protective and cross–protective efficacy	[17, 38]
	Chicken	Safety, attenuation	[17, 38]
Len–Vn (H5N2)	Ferrets	Safety, attenuation, cross–protective efficacy	[17]
	Chicken	Safety, attenuation	[17]
Len–Vn/rg (H5N1)	Mice	Infectivity in respiratory tract; immunogenicity, protective and cross–protective efficacy	[30, 31]
	Ferrets	Attenuation, immunogenicity, cross–protective efficacy	[17]
	Chicken	Safety, attenuation	[17, 38]
Len–Eg/rg (H5N1)	Mice	Infectivity in respiratory tract; immunogenicity, protective and cross–protective efficacy	[31]

**Table 3:** Results of clinical trials of H5–LAIVs against potentially pandemic influenza viruses in healthy vaccinated adults after the first and the second doses.

H5–LAIV	Dose	Number (%) of Positive Subjects of Total Number Vaccinees							Refs.
		Adverse Reactions		Virus Shedding		Genetic Stability	Immunogenicity^2^		
		Local^1^	Systemic^2^	PCR^3^	Culture^4^	Stability of *att* Mutations	Any Antibody Response^5^	Any Cell Mediated Response^6^	
Len–dP	1	8/20 (40)	0/20 (0)	Not tested	11/20 (55)	Confirmed	16/42 (38)	4/10 (40)	[39, 48]
	2	0/20 (0)	0/20 (0)		14/20 (70)		30/42 (71)	6/10 (60)	
Len–tT	1	2/30 (7)	12/30 (40)	28/30 (93)	10/30 (33)	Confirmed	8/29 (28)	13/29 (45)	[40, 42]
	2	1/29 (3)	6/29 (20)	21/29 (72)	6/29 (21)		23/29 (79)	12/29 (41)	

^1^Local adverse reactions (nasal congestion, sneezing, runny nose, hyperemia of the fauces and arches, catarrhal nasopharynx).

^2^Systemic adverse reactions (fever, chills, fatigue, sore throat, headache, muscle and joint aches, nausea, vomiting, cough).

^3^Detection of vaccine virus in nasal wash/swab samples by RT–PCR.

^4^Isolation of vaccine virus from nasal wash/swab samples in embryonated chicken eggs.

^5^Antibody immune responses (serum IgG, serum IgA, mucosal IgA).

^6^Cell mediated immune responses (virus–specific CD4^+^IFNγ^+^ and CD8^+^IFNγ^+^).

## LIST OF ABBREVIATIONS

*att*: = attenuation
*ca*: = cold–adapted
H5–LAIV: = live attenuated influenza vaccine against H5N1 avian influenza viruses
HPAIV: = Highly Pathogenic Influenza Virus
IIV: = Inactivated Influenza Vaccine
LAIV: = Live Attenuated Influenza Vaccine
Len–dP: = A/17/duck/Potsdam/86/92 (H5N2)
Len–Eg/rg: = caEG321–Len17rg (H5N1)
Len–MDV: = A/Leningrad/134/17/57 (H2N2) master donor virus
Len–tT: = А/17/turkey/Turkey/05/133 (H5N2)
Len–Vn: = A/17/Vietnam/04/65107 (H5N2)
Len–Vn/rg: = caVN1203–Len17rg (H5N1)
MDV: = Master Donor Virus
rg (RG): = reverse genetics
*ts*: = temperature sensitive
WHO: = World Health Organization
